# Supplementary material for: Defective metabolic programming impairs early neuronal morphogenesis in neural cultures and an organoid model of Leigh syndrome
Source: Nat Commun. 2021 Mar 26;12:1929. doi: 10.1038/s41467-021-22117-z (PMC7997884; doi:10.1038/s41467-021-22117-z)
Supplement: Supplementary file 1 — Supplementary Information [file 41467_2021_22117_MOESM1_ESM.pdf]

SUPPLEMENTARY INFORMATION

Defective metabolic programming impairs early neuronal morphogenesis in neural cultures and an organoid model of Leigh syndrome

Inak et al.

Manuscript correspondence:

Alessandro Prigione, alessandro.prigione@hhu.de

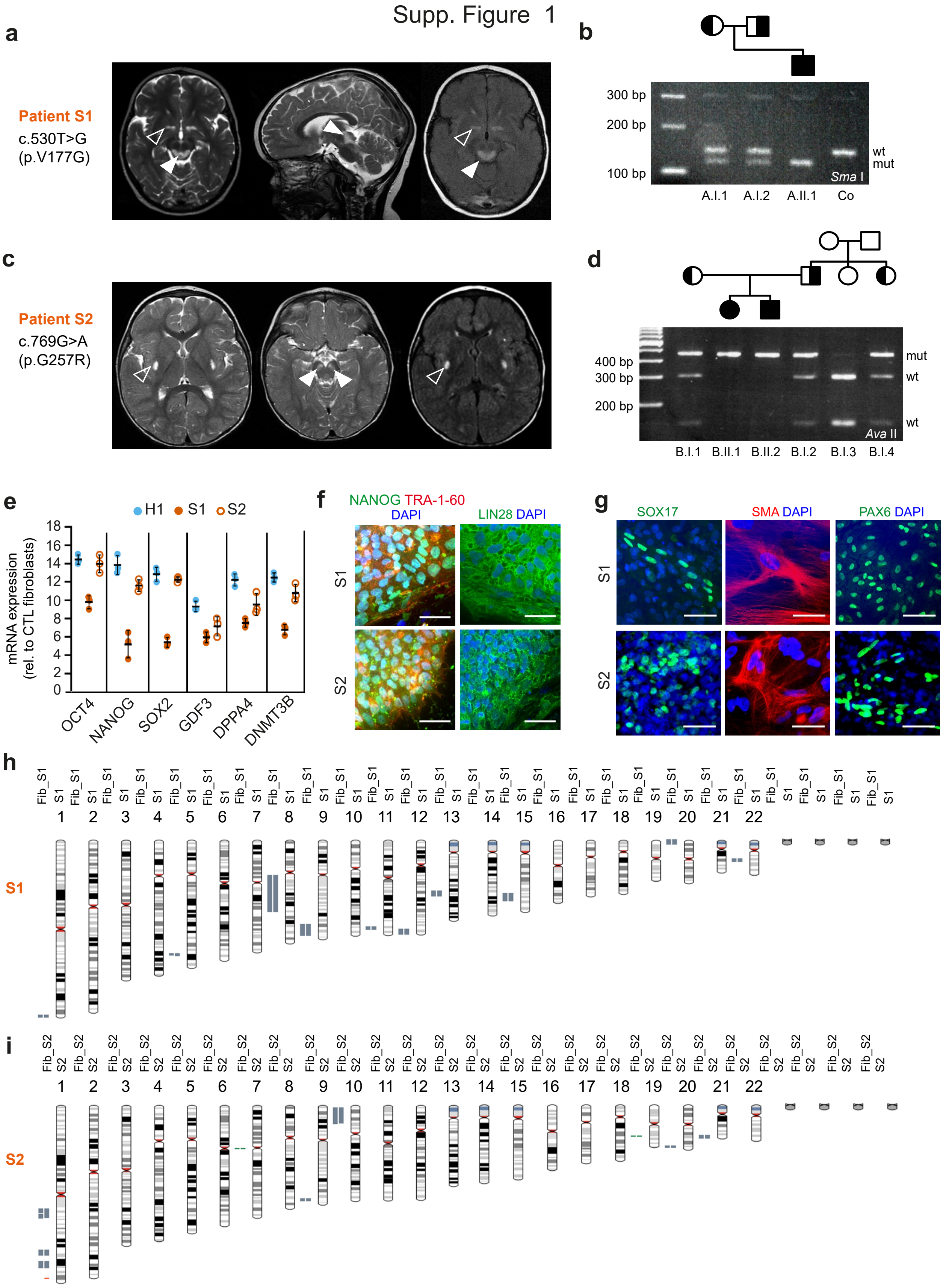

Supp. Figure 1
a
Patient S1
c.530T>G
(p.V177G)
b
300 bp
200 bp
100 bp
wt
mut
Sma I
A.I.1
A.I.2
A.II.1
Co
c
Patient S2
c.769G>A
(p.G257R)
d
400 bp
300 bp
200 bp
mut
wt
wt
Ava II
B.I.1
B.II.1
B.II.2
B.I.2
B.I.3
B.I.4
e
H1 S1 S2
mRNA expression
(rel. to CTL fibroblasts)
OCT4
NANOG
SOX2
GDF3
DPPA4
DNMT3B
f
NANOG TRA-1-60 DAPI
LIN28 DAPI
S1
S2
g
SOX17
SMA DAPI
PAX6 DAPI
S1
S2
h
S1
i
S2

Supplementary Fig. 1: Generation of SURF1 iPSCs (related to Fig. 1).

a Cranial magnetic resonance imaging (MRI) of patient S1 at 2.3 years of age. Open arrowheads depict T2-signal intense lesions in the basal ganglia as the pathological hallmark of LS. White arrowheads point at brainstem lesions compromising the *formatio reticularis*. The lesions of the basal ganglia are further accentuated in the FLAIR sequences (right). **b** Pedigree of patient 1 (S1) family showing the segregation of the LS phenotype with homozygosity for the c.530T>G mutation in the S1 family (A.II.1). We assessed the mutation via PIRA using the restriction enzyme *SmaI* (n=3 independent experiments). **c** Cranial MRI of patient 2 (S2) at 1.7 years of age. Open arrowheads depict T2-signal intense lesions in the basal ganglia. White arrowheads point at lesions in the *substantia nigra*. The lesions are further accentuated in the FLAIR sequences (right). **d** Pedigree of S2 family showing the segregation between the LS phenotype with homozygosity for the c.769G>A mutation in the family of S2 sibling (B.II.1) and S2 patient (B.II.2). We assessed the mutation via PIRA using the restriction enzyme *AvaII* (n=3 independent experiments). **e** QRT-PCR of pluripotency markers in hESCs (H1) and SURF1 iPSCs (SURF1_Mut: S1, S2) relative to control fibroblasts (C1) (normalized to *ACTB*; mean +/- s.d.; n=3 independent experiments). **f** Immunostainings showing pluripotency-associated protein markers NANOG, TRA-1-60, and LIN28 in SURF1 iPSCs (SURF1_Mut: S1, S2) (n=2 independent experiments). Scale bar: 50 µm. **g** Embryoid body (EB)-based *in vitro* differentiation of SURF1 iPSCs (SURF1_Mut: S1, S2). Three germ layers represented by SOX17 for endoderm, smooth muscle actin (SMA) for mesoderm, and PAX6 for ectoderm. We counterstained nuclei using Hoechst (n=2 independent experiments). Scale bar: 50 µm. **h-i** Karyotype analysis of SURF1 iPSCs (SURF1_Mut: S1, S2) compared to the original parental fibroblasts (Fib_S1 and Fib_S2).

Supp. Figure 2

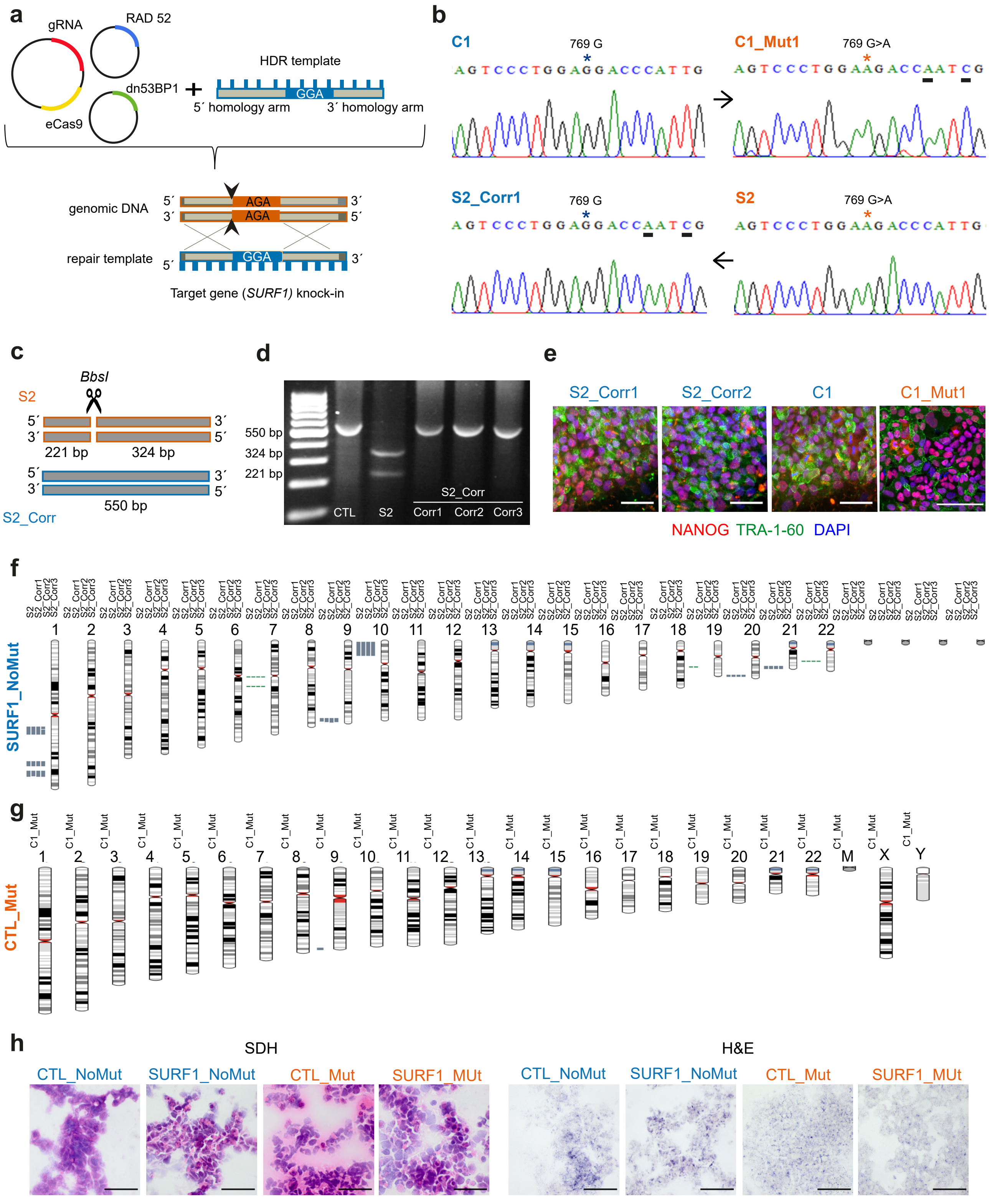

Supplementary Fig. 2: Genome engineering of SURF1 iPSCs and CTL iPSCs (related to Fig. 1).

a CRISPR/eCas9 knock-in strategy for *SURF1* mutation c.769G>A. Homologous direct repair (HDR) template (in blue) targeting the *SURF1* genomic region around 769G>A (in orange), with plasmids (i) pU6(BbsI)-CAG-eCas9-venus-bpA, 10,146 bp vector coding for a specific designed gRNA (red) and for the endonuclease Cas9 (yellow), (ii) 614.pCAG-hRad52-EF1BFP, 8,207 bp plasmid coding for RAD 52 (blue), (iii) 602.pCAG-i53-EF1BFP, 7,175 bp vector coding for dn53BP1 (green). **b** Electropherograms showing a portion of the *SURF1* sequence for CTL (CTL_NoMut: C1), CTL_Mut (C1_Mut1), SURF1_Mut (S2), and SURF1_NoMut (S2_Corr1). Blue stars: wild-type base pair; orange stars: mutant base pair; black underlines: artificially introduced silent mutations. **c-d** Visualization of successful knock-in events by cutting PCR products (550 bp long in healthy conditions) using restriction enzyme *BbsI*. In the presence of mutation c.769 G>A, the enzyme generated two fragments (221 bp + 324 bp). SURF1_NoMut clones S2_Corr1, S2_Corr2 and S2_Corr3 showed only one band of the same sizes as CTL_NoMut (C1) (n=3 independent experiments). **e** Pluripotency markers expression in genome-edited iPSC lines: SURF1_NoMut (S2_Corr1, S2_Corr2) engineered from SURF1_Mut (line S2), and CTL_Mut (C1_Mut1) engineered from control CTL_NoMut (line C1) (n=3 independent experiments). Nuclei were counterstained with Hoechst. Scale bar: 50 µm. **f** Karyotype analysis of isogenic control lines SURF1_NoMut (S2_Corr1, S2_Corr2, S2-Corr3) derived from SURF1 iPSC line S2 (n=2 independent experiments). **g** Karyotype analysis of isogenic mutant CTL_Mut (C1_Mut1) derived from CTL iPSC line C1 (n=2 independent experiments). **h** Mitochondrial enzyme activity for RC complex II succinate dehydrogenase (SDH) in CTL NPCs (CTL_NoMut: C1; SURF1_NoMut: S2_Corr1) and SURF1 NPCs (SURF1_Mut: S2; CTL_Mut: C1_Mut1). Reproduced in CTL NPCs (CTL_NoMut: C2, C3; SURF1_NoMut: S2_Corr2) and SURF1 NPCs (SURF1_Mut: S1) (n=3 independent experiments). We used haematoxylin and eosin (H&E) as a control staining. Scale bar: 500 nm.

Supp. Figure 3

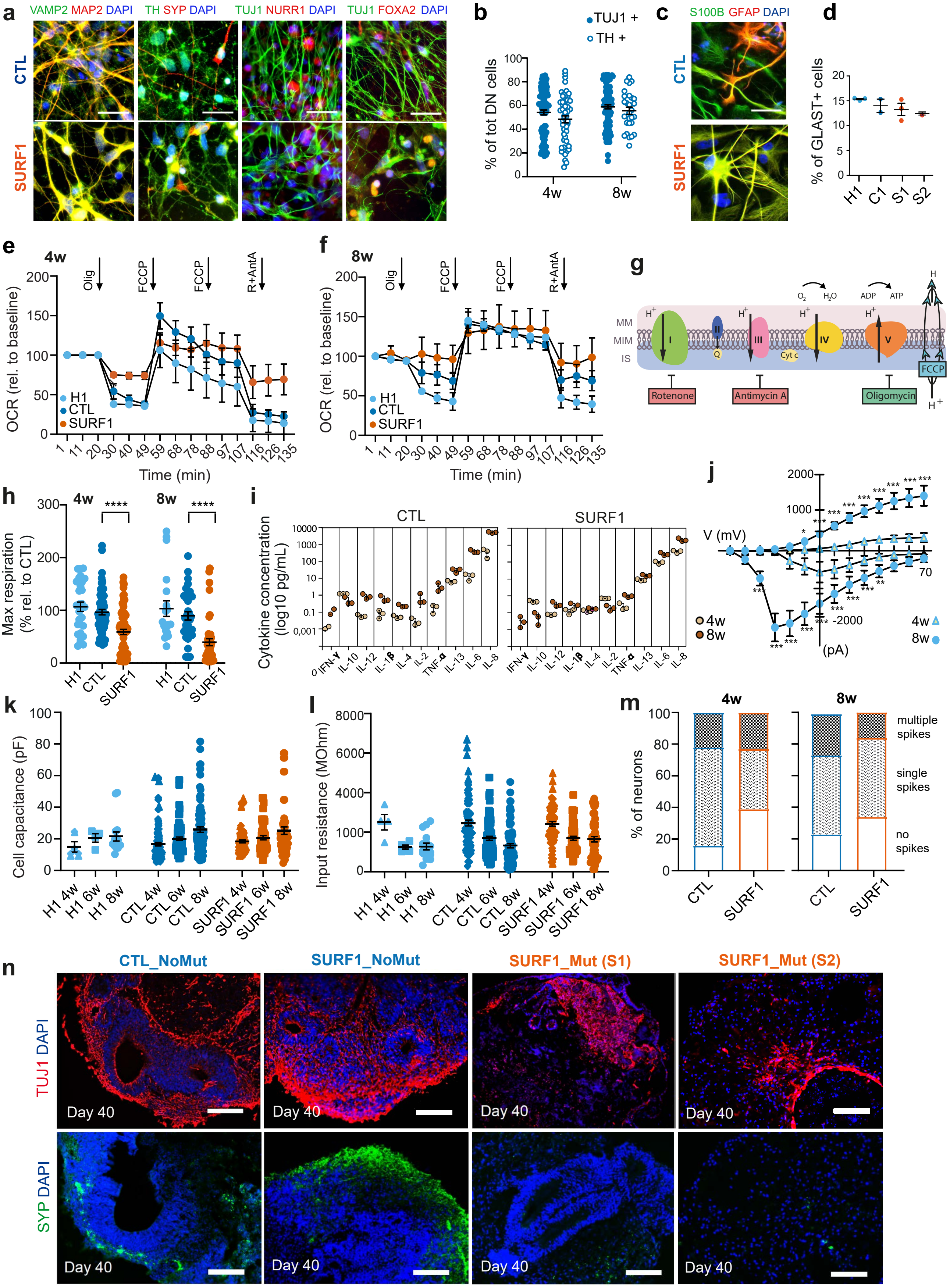

Supplementary Fig. 3: Characterization of 2D and 3D neuronal models of *SURF1* mutations (related to Fig. 2).

a 8w DNs from CTL (CTL_NoMut: C2) and SURF1 (SURF1_Mut: S2) (n=3 independent experiments). Scale bar: 50 µm. **b** Percentage of TUJ1+ (empty box) and TH+ neurons (dotted box) in 4w DNs and 8w DNs from CTL (CTL_NoMut: C1, C2, C3; SURF1_NoMut: S2_Corr1, S2_Corr2) (mean +/- s.e.m.; n=15 biological replicates (dots) per line over 3 independent experiments). **c** Glial cells in 8w DNs from CTL (CTL_NoMut: C1) and SURF1 (SURF1_Mut: S2) (n=3 independent experiments). Scale bar: 50 µm. **d** MACS-based quantification of astrocyte marker GLAST in 8w DNs from hESCs (H1), CTL (CTL_NoMut: C1), and SURF1 (SURF1_Mut: S1, S2) (mean +/- s.e.m.; n=3 independent experiments). **e-f** OCR of 4w DNs and 8w DNs from hESCs (H1), CTL (CTL_NoMut: C1, C2, C3; SURF1_NoMut: S2_Corr1), and SURF1 (SURF1_Mut: S1, S2; CTL_Mut: C1_Mut1) (mean +/- s.e.m.; n=3 independent experiments). **g** Mode of action of the mitochondrial inhibitors during bioenergetic profiling. **h** Maximum respiration rate of 4w DNs and 8w DNs from hESCs (H1), CTL (CTL_NoMut: C1, C2, C3; SURF1_NoMut: S2_Corr1) and SURF1 (SURF1_Mut: S1, S2; CTL_Mut: C1_Mut1) (mean +/- s.e.m.; n=15 biological replicates (dots) per line over 3 independent experiments; ****p<0.0001 CTL vs. SURF1; Two-sided Mann-Whitney U test). **i** Extracellular cytokines in 4w DNs (light brown) and 8w DNs (dark brown) from CTL (CTL_NoMut: C1) and SURF1 (SURF1_Mut: S2). Reproduced in hESCs (H1), CTL (CTL_NoMut: C2, C3), and SURF1 (SURF1_Mut: S1) (mean +/- s.e.m.; n=3 independent experiments). **j** Sodium and potassium currents in 4w DNs and 8w DNs from hESCs (H1) (mean +/- s.d.; n=40 cells per line over 2 independent experiments; *p<0.05, **p<0.01, ***p<0.001 8w vs. 4w; Two-sided Mann-Whitney U test). **k-l** Cell capacitance (pF) and input resistance (MOhm) in 4w DNs, 6w DNs, and 8w DNs from hESCs (H1), CTL (CTL_NoMut: C1, C2, C3; SURF1_NoMut: S2_Corr1), and SURF1 (SURF1_Mut: S1, S2) (mean +/- s.e.m.; n=6 cells per line per condition over 2 independent experiments). **m** Percentage of firing neurons (mean) in 4w DNs and 8w DNs from CTL (CTL_NoMut: C1, C2, C3; SURF1_NoMut: S2_Corr1), and SURF1 (SURF1_NoMut: S1, S2) (mean +/- s.e.m.; n=40 cells per line over 2 independent experiments). **n** D40 cerebral organoids from CTL (CTL_NoMut: C1; SURF1_NoMut: S2_Corr1) and SURF1 (SURF1_Mut: S1, S2). Reproduced in CTL (CTL_NoMut: C2) and SURF1 (CTL_Mut: C1_Mut1) (3-8 organoids per line per experiment, n=3 independent experiments). Scale bar: 100 µm.

Supp. Figure 4

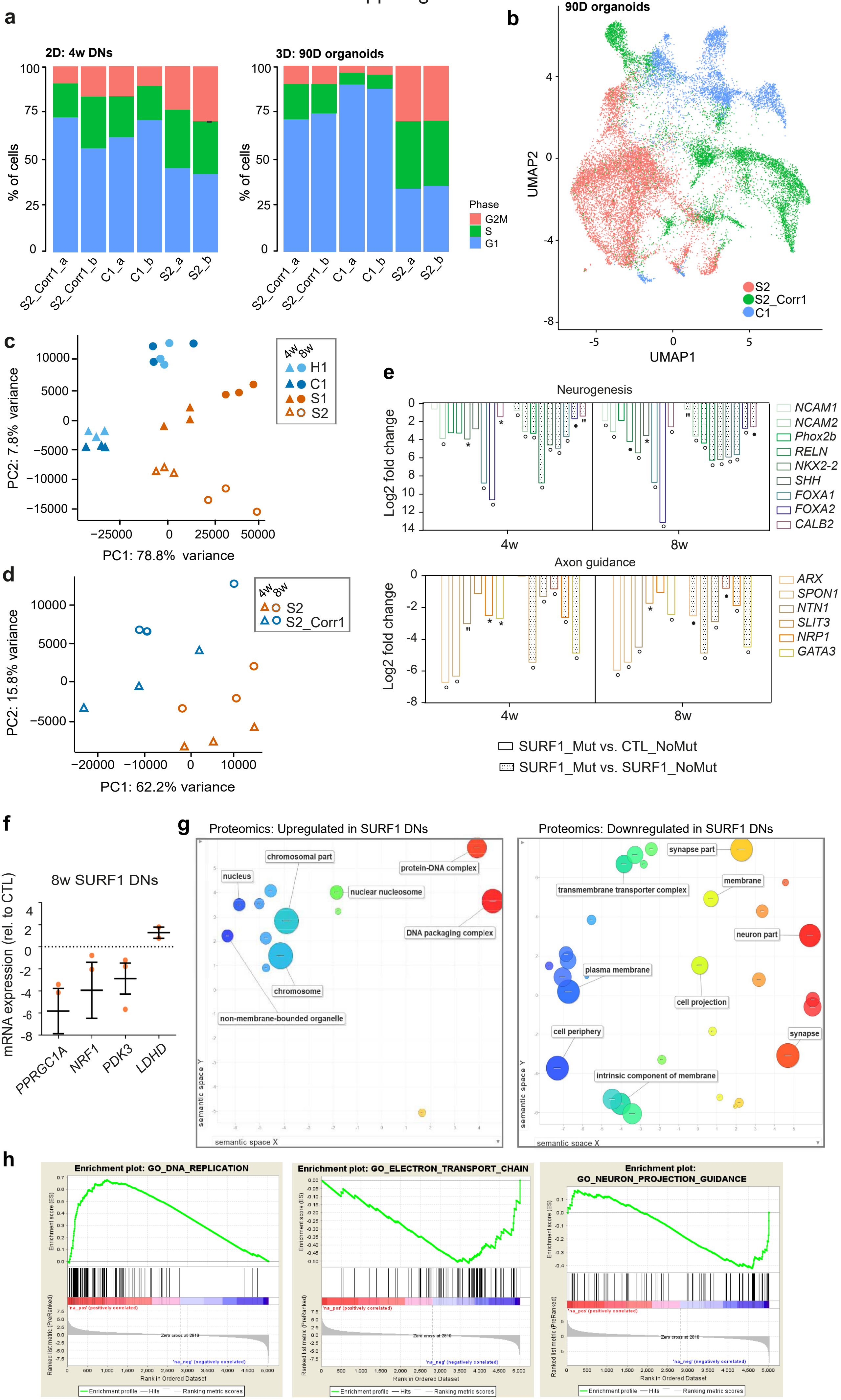

Supplementary Fig. 4: Single-cell transcriptomics and multi-omics integration of SURF1 neuronal cultures (related to Fig. 3 and Fig. 4).

a Cell cycle distribution in 4w DNs from CTL (CTL_NoMut: C1; SURF1_NoMut: S2_Corr1) and SURF1 (SURF1_Mut: S2), and D90 brain organoids from CTL (CTL_NoMut: C1; SURF1_NoMut: S2_Corr1) and SURF1 (SURF1_Mut: S2) (3-8 organoids per line per experiment; n=2 independent experiments indicated with "a" and "b"). **b** UMAP plot depicting the distribution of D90 organoids from CTL (CTL_NoMut: C1; SURF1_NoMut: S2_Corr1) and SURF1 (SURF1_Mut: S2) (3-8 organoids per line per experiment; n=2 independent experiments). **c** Principal component (PC) analysis of 4w DNs and 8w DNs from hESCs (H1), CTL (CTL_NoMut: C1), and SURF1 (SURF1_Mut: S1, S2) based on mRNA-sequencing (n=3 independent experiments) (see Supplementary Data 10). **d** PC analysis of 4w DNs and 8w DNs from CTL (SURF1_NoMut: S2_Corr1) and SURF1 (SURF1_Mut: S2) based on total RNA-sequencing (n=3 independent experiments) (see Supplementary Data 5 for 8w DNs and Supplementary Data 9 for 4w DNs). **e** Differentially regulated genes associated with neurogenesis and axon guidance based on RNA-seq analysis in 4w DNs and 8w DNs from SURF1_Mut (S1, S2) compared to CTL_NoMut (C1) (see Supplementary Data 10) and in 4w and 8w DNs from SURF1_Mut (S2) compared to SURF1_NoMut (S2_Corr1) (see Supplementary Data 5) (n=3 independent experiments; *p<0.05, ´´p<0.01, οp<0.001, ●p<0.0001 CTL vs. SURF1; Two-tailed Wald test with Benjamini-Hochberg correction for multiple comparisons; FDR<0.05). **f** QRT-PCR of genes regulating mitochondrial bioenergetics (*PPRGC1A, NRF1*, and *PDK3*) and glycolysis (*LDHD*) in 8w DNs from CTL (SURF1_NoMut: S2_Corr1) and SURF1 (SURF1_Mut: S2) (normalized to *ACTB*; mean +/- s.d; n=3 independent experiments). **g** Rovigo representation of proteomics of 8w DNs from CTL (SURF1_NoMut: S2_Corr1) and SURF1 (SURF1_Mut: S2) based on GO terms related to "cell compartment". Left: upregulated cell compartment in SURF1 vs. CTL; right: downregulated cell compartment in SURF1 vs. CTL (n=3 independent experiments). **h** Gene set enrichment analysis (GSEA) of proteomics dataset. Examples of pathways upregulated (left) and downregulated (middle and right) in 8w DNs from SURF1 (SURF1_Mut: S2) compared to CTL (SURF1_NoMut: S2_Corr1) (n=3 independent experiments).

Supp. Figure 5

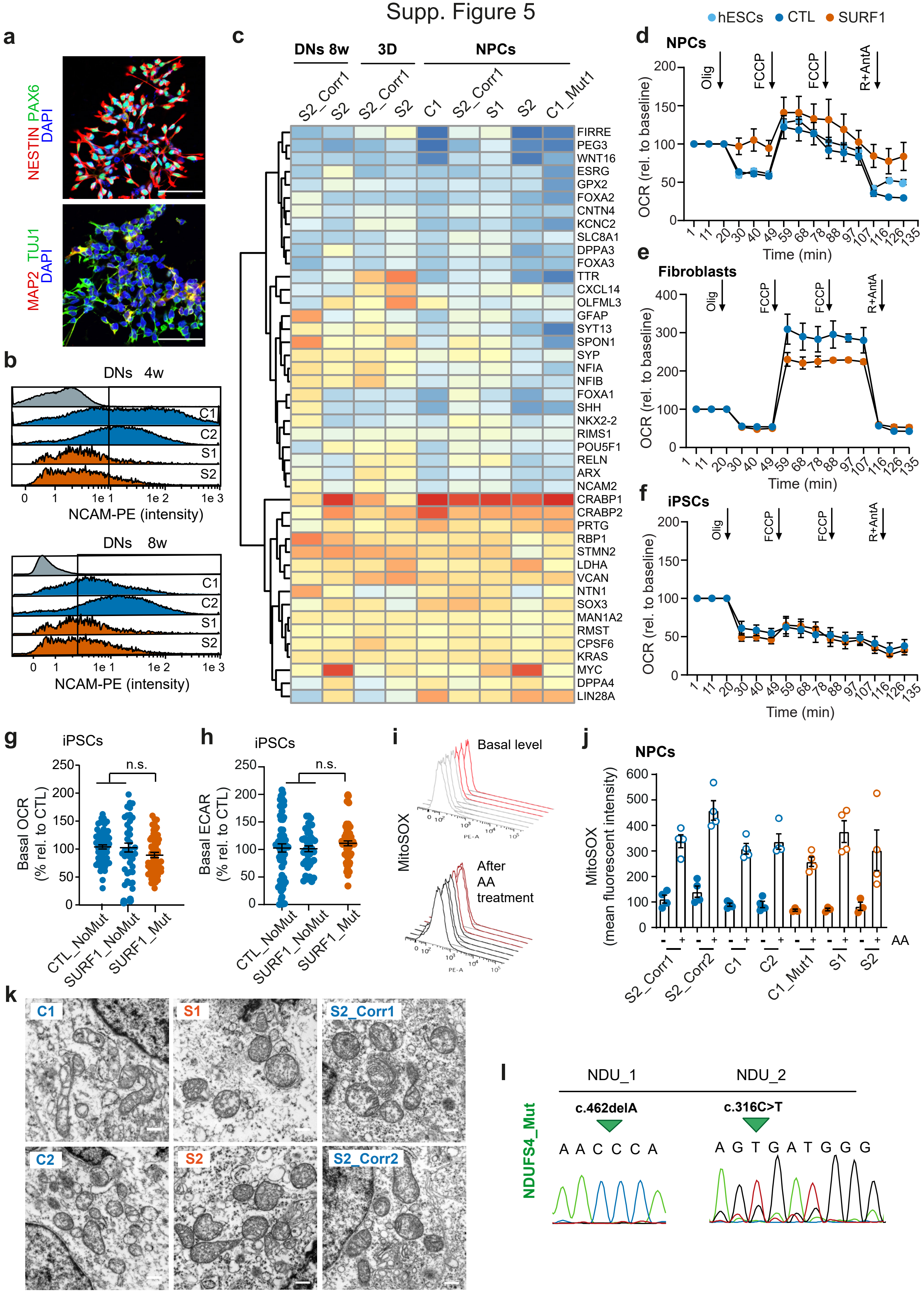

Supplementary Fig. 5: Phenotypic characterization of SURF1 NPCs (related to Fig. 5).

a SURF1 NPCs (SURF1_Mut: S2) showing NPC markers (NESTIN, PAX6), and neuronal markers (TUJ1, MAP2) (n=3 independent experiments). Scale bar: 50 µm. **b** MACS-sorted NCAM+ cells in 4w DNs and 8w DNs from CTL (CTL_NoMut: C1, C2) and SURF1 (SURF1_Mut: S1, S2). Grey: NCAM negative sorted cells; x-axis: fluorescence intensity; y-axis: cell number (n=3 independent experiments). **c** Heatmap of Nanostring expression analysis of 8w DNs from CTL (SURF1_NoMut: S2_Corr1) and SURF1 (SURF1_Mut: S2), 90D brain organoids from CTL (SURF1_NoMut: S2_Corr1) and SURF1 (SURF1_Mut: S2), and NPCs from CTL (CTL_NoMut: C1; SURF1_NoMut: S2_Corr1) and SURF1 (SURF1_Mut: S1, S2; CTL_Mut: C1_Mut1) (3-8 organoids per line per experiment; n=3 independent experiments). Blue: low expression level; red: high expression level. **d** OCR of NPCs from hESCs (H1), CTL (CTL_NoMut: C1, C2, C3; SURF1_NoMut: S2_Corr1), and SURF1 (SURF1_Mut: S1, S2; CTL_Mut: C1_Mut1) (mean +/- s.e.m.; n=25 biological replicates per line over 3 independent experiments). **e** OCR of fibroblasts from CTL (CTL_NoMut: C2, C3) and SURF1 (SURF1_Mut: S1, S2) (mean +/- s.e.m.; n=25 biological replicates per line over 2 independent experiments). **f** OCR of iPSCs from CTL (SURF1_NoMut: S2_Corr1, S2_Corr2) and SURF1 (SURF1_Mut: S2) (mean +/- s.e.m.; n=25 biological replicates per line over 2 independent experiments). **g-h** OCR and ECAR of iPSCs from CTL (CTL_NoMut: C1; SURF1_NoMut: S2_Corr1, S2_Corr2) and SURF1 (SURF1_Mut: S2) (mean +/- s.e.m.; n=25 biological replicates (dots) per line over 2 independent experiments; n.s.=not significant CTL vs. SURF1; Two-sided Mann-Whitney U test). **i-j** FACS-based prolife (left) and quantification (right) of mean fluorescence intensity of mitochondrial ROS detector MitoSOX in NPCs from CTL (CTL_NoMut: C1, C2; SURF1_NoMut: S2_Corr1, S2_Corr2) and SURF1 (SURF1_Mut: S1, S2; CTL_Mut: C1_Mut1). We performed the experiments at the basal level and after 1h treatment with 3 µM antimycin A (AA) to stimulate ROS (mean +/- s.d.; n=4 independent experiments). **k** Mitochondrial morphology by electron microscopy in NPCs from CTL (CTL_NoMut: C1, C2; SURF1_NoMut: S2_Corr1, S2_Corr2) and SURF1 (SURF1_Mut: S1, S2) (n=2 independent experiments). Scale bar: 500 nm. **l** Electropherograms o iPSCs NDUFS4_Mut (NDU_1, NDU_2) derived from two LS patients carrying mutations in CI gene *NDUFS4*.

Supp. Figure 6

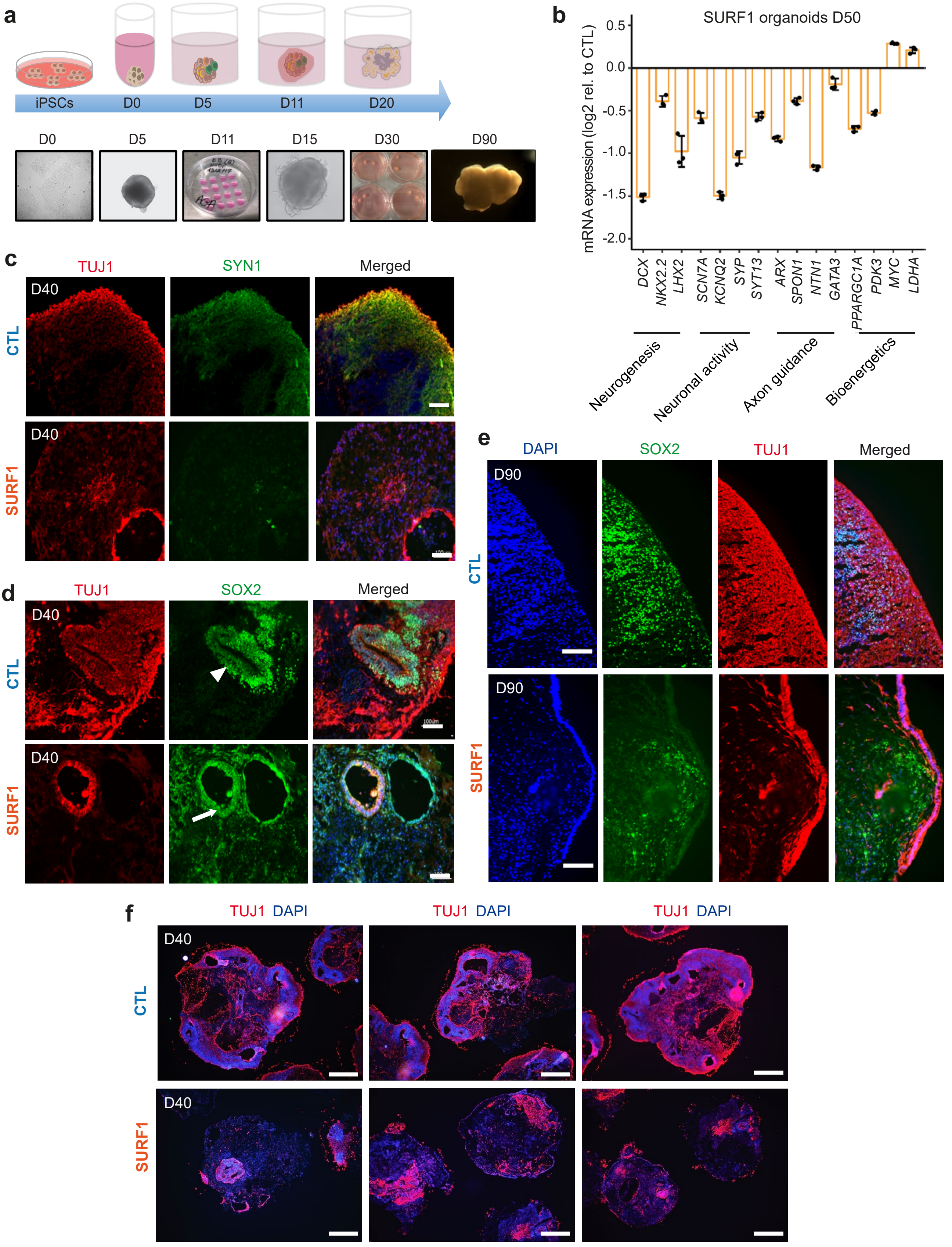

Supplementary Fig. 6: Cytoarchitecture and neural progenitor organization in SURF1 brain organoids (related to Fig. 6).

a Cerebral organoid maturation from the day of embedding into Matrigel (day 0). Scale bars: D0=500 µm, D10=200 µm, D40=200 µm. **b** QRT-PCR-based expression of genes associated with neurogenesis, neuronal activity, axon guidance, and bioenergetics in D50 cerebral organoids from CTL (SURF1_NoMut: S2_Corr1) and SURF1 (SURF1_Mut: S2) (normalized over *GAPDH* expression; mean +/- s.d.; 3-8 organoids per line per experiment; n=3 independent experiments). **c** Representative immunofluorescence images of D40 cerebral organoids from CTL (SURF1_NoMut: S2_Corr1) and SURF1 (SURF1_Mut: S2) for neuronal marker TUJ1 and synaptic marker SYN1 (3-8 organoids per line per experiment; n=3 independent experiments Scale bar: 100 µm. **d** D40 cerebral organoids from CTL (SURF1_NoMut: S2_Corr1) and SURF1 (SURF1_Mut: S2). White arrowhead: SOX2+ neuroepithelial layering; white arrow: thinning and disruption of SOX2+ neuroepithelial layering. Reproduced in CTL (CTL_NoMut: C2; SURF1_NoMut; S2_Corr1) and SURF1 (SURF1_Mut: S2) (3-8 organoids per line per experiment; n=2 independent experiments Scale bar: 100 µm. **e** D90 cerebral organoids from CTL (SURF1_NoMut: S2_Corr1) and SURF1 (SURF1_Mut: S1) for progenitor marker SOX2 and neuronal marker TUJ1 (3-8 organoids per line per experiment; n=2 independent experiments) Scale bar: 100 µm. **f** D40 organoids from CTL (CTL_NoMut: C1) and SURF1 (SURF1_Mut: S2) showing the differences in size across replicates. Reproduced in CTL (CTL_NoMut: C2) and SURF1 (SURF1_Mut: S1; CTL_Mut: C1_Mut1) (3-8 organoids per line per experiment, n=3 independent experiments). Scale bar: 500 µm.

Supp. Figure 7

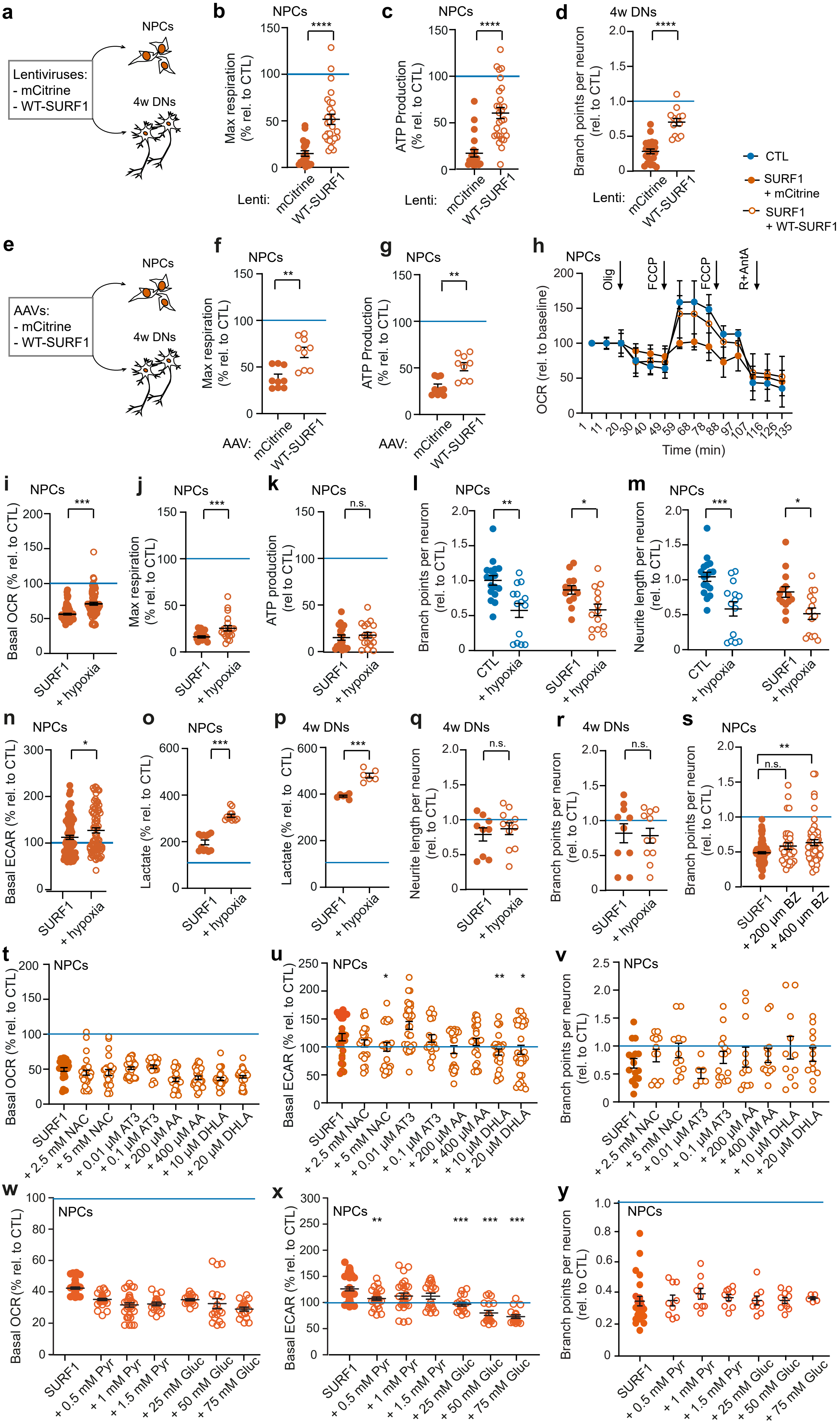

Supplementary Fig. 7: Treatment strategies in SURF1 neural cells (related to Fig. 7).

a Scheme of lentiviral delivery. **b-c** Bioenergetics in SURF1 NPCs (SURF1_Mut: S1, S2) after lentiviral delivery (mean +/- s.e.m.; n=10 biological replicates (dots) per line over 2 independent experiments; ****p<0.0001; Two-sided Mann-Whitney U test). **d** Morphogenesis in 4w DNs from SURF1 (SURF1_Mut: S1, S2) after lentiviral delivery (mean +/- s.e.m.; n=10 biological replicates (dots) per line over 2 independent experiments; ****p<0.00=1; Two-sided Mann-Whitney U test). **e** Scheme of AAV delivery. **f-g** Bioenergetics in SURF1 NPCs (SURF1_Mut: S1, S2) after AAV delivery (mean +/- s.e.m.; n=4 biological replicates (dots) per line over 2 independent experiments; **p<0.001; Two-sided Mann-Whitney U test). **h** OCR in CTL NPCs (CTL_NoMut: C1; SURF1_NoMut: S2_Corr1) and SURF1 NPCs (SURF1_Mut: S1, S2) after AAV delivery (mean +/- s.e.m.; n=15 biological replicates per line over 2 independent experiments). **i-k** Bioenergetics of SURF1 NPCs (SURF1_Mut: S1, S2) under normoxia or hypoxia (mean +/- s.e.m.; n=6 biological replicates (dots) per line over 3 independent experiments; n.s.=not significant, ***p<0.001; Two-sided Mann-Whitney U test). **l-m** Morphogenesis in NPCs from CTL (CTL_NoMut: C1; SURF1_NoMut: S2_Corr1) and SURF1 (SURF1_Mut: S1, S2) under normoxia or hypoxia (mean +/- s.e.m.; n=7 biological replicates (dots) per line over 2 independent experiments; *p<0.05, **p=0.0016, ****p<0.0001; Two-sided Mann-Whitney U test). **n-o** OCR and lactate release in SURF1 NPCs (SURF1_Mut: S1, S2) under normoxia or hypoxia (mean +/- s.e.m.; n=5 biological replicates (dots) per line over 2 independent experiments; *p=0.0416, ***p<0.001; Two-sided Mann-Whitney U test). **p-r** Lactate release and morphogenesis in 4w SURF1 DNs (SURF1_Mut: S1, S2) under normoxia or hypoxia (mean +/- s.e.m.; n=3 biological replicates per line over 3 independent experiments; ***p<0.001, n.s.=not significant; Two-sided Mann-Whitney U test). **s** Morphogenesis in SURF1 NPCs (SURF1_Mut: S1, S2; CTL_Mut: C1_Mut1) with BZ compared to UT (mean +/- s.e.m.; n=15 biological replicates (dots) per line over 3 independent experiments; n.s.=not significant, **p=0.0029; Two-sided Mann-Whitney U test). **t-y** Bioenergetics and morphogenesis of SURF1 NPCs (SURF1_Mut: S1, S2) treated with n-acetyl l-cysteine (NAC), a-tocotrienol (AT3), ascorbic acid (AA), dihydrolipoic acid (DHLA), or with pyruvate or glucose addition (mean +/- s.e.m.; n=3 biological replicates (dots) per line over 2 independent experiments; *p<0.05, **p<0.01, ***p<0.001; Two-sided Mann-Whitney U test). Horizontal blue lines in all panels: average values CTL (CTL_NoMut: C1; SURF1_NoMut: S2_Corr1).

Supp. Figure 8

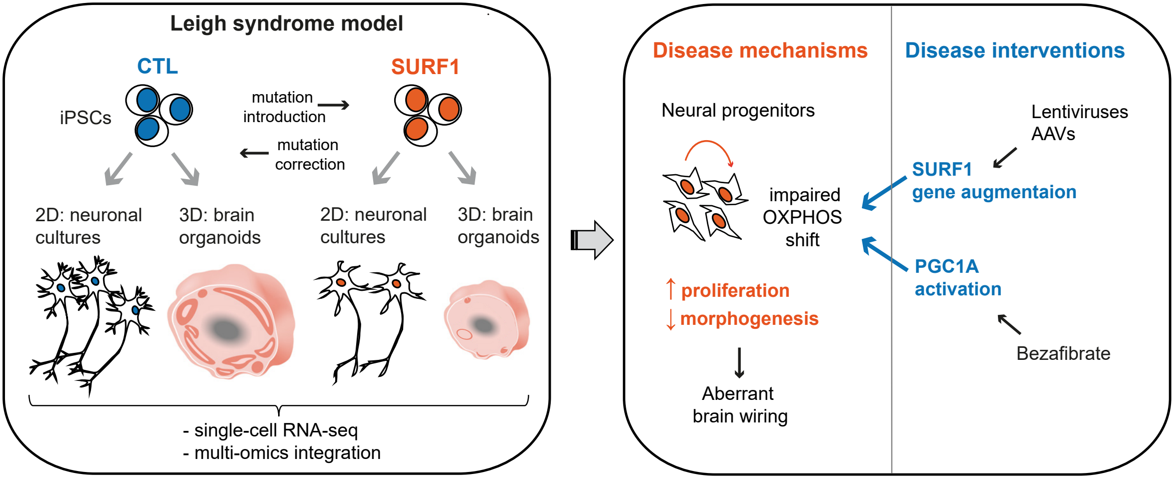

Supplementary Fig. 8: Schematics summarizing the findings of the current study.
