# Supplementary material for: Defective metabolic programming impairs early neuronal morphogenesis in neural cultures and an organoid model of Leigh syndrome
Source: Nat Commun. 2021 Mar 26;12:1929. doi: 10.1038/s41467-021-22117-z (PMC7997884; doi:10.1038/s41467-021-22117-z)
Supplement: Supplementary file 3 — Description of Additional Supplementary Files [file 41467_2021_22117_MOESM3_ESM.pdf]

Description of Additional Supplementary Files

File Name: Supplementary Data 1.

Description: Off-target analysis of SURF1_NoMut (S2_Corr1) compared to SURF1_Mut (S2) (related to Fig. 1).

File Name: Supplementary Data 2.

Description: Summary of experiments carried out with brain organoids (related to Fig. 2).

File Name: Supplementary Data 3.

Description: Single-cell RNA-sequencing: quality controls and genes present within the clusters identified in 4w DNs (CTL_NoMut: C1; SURF1_NoMut: S2_Corr1; SURF1_Mut: S2) and D90 brain organoids (SURF1_NoMut: S2_Corr1; SURF1_Mut: S2) (related to Fig. 3).

File Name: Supplementary Data 4.

Description: Pathways enriched in community 1, community 2, and community 3 identified following multi-omics integration of 8w DNs from CTL (SURF1_NoMut: S2_Corr1) and SURF1 (SURF1_Mut: S2) (related to Fig. 4).

File Name: Supplementary Data 5.

Description: Total RNA-sequencing: differentially expressed genes and pathways in 8w DNs from CTL (SURF1_NoMut: S2_Corr1) and SURF1 (SURF1_Mut: S2) (related to Fig. 4) (two-tailed Wald test with Benjamini-Hochberg correction for multiple comparisons; FDR<0.05).

File Name: Supplementary Data 6.

Description: Proteomics: differentially expressed proteins in 8w DNs from CTL (SURF1_NoMut: S2_Corr1) and SURF1 (SURF1_Mut: S2) (related to Figure 4) (two-tailed t-test with Benjamini-Hochberg correction for multiple comparisons; FDR<0.05).

File Name: Supplementary Data 7.

Description: Proteomics: Differentially expressed pathways (GO biological processes) in 8w DNs from CTL (SURF1_NoMut: S2_Corr1) and SURF1 (SURF1_Mut: S2) (related to Fig. 4) (two-tailed t-test with Benjamini-Hochberg correction for multiple comparisons; FDR<0.05).

File Name: Supplementary Data 8.
Description: Metabolomics: differentially contained metabolites in 8w DNs from CTL (SURF1_NoMut: S2_Corr1) and SURF1 (SURF1_Mut: S2) (related to Fig. 4) (two-tailed t-test with Benjamini-Hochberg correction for multiple testing; FDR<0.05).

File Name: Supplementary Data 9.
Description: Total RNA-sequencing: differentially expressed genes and pathways 4w DNs from CTL (SURF1_NoMut: S2_Corr1) and SURF1 (SURF1_Mut: S2) (related to Fig. 4) (two-tailed Wald test with Benjamini-Hochberg correction for multiple comparisons; FDR<0.05).

File Name: Supplementary Data 10.
Description: mRNA-sequencing: differentially expressed genes and pathways in 4w DNs and 8w DNs from hESCs (H1), CTL (CTL_NoMut: C1), and SURF1 (SURF1_Mut: S1. S2) (related to Fig. 4) (two-tailed Wald test with Benjamini-Hochberg correction for multiple comparisons; FDR<0.05).

File Name: Supplementary Data 11.
Description: Total RNA-sequencing: differentially expressed genes and pathways in D90 cerebral organoids from CTL (SURF1_NoMut: S2_Corr1) and SURF1 (SURF1_Mut: S2) (related to Fig. 6) (two-tailed Wald test with Benjamini-Hochberg correction for multiple comparisons; FDR<0.05).

File Name: Supplementary Data 12.
Description: List of sequences and primers for QRT-PCR, PIRA PCR, and CRISRP/eCas9 genome editing.

File Name: Supplementary Data 13.
Description: Details regarding all iPSC lines and their use in all individual figures.
