# Supplementary material for: Defective metabolic programming impairs early neuronal morphogenesis in neural cultures and an organoid model of Leigh syndrome
Source: Nat Commun. 2021 Mar 26;12:1929. doi: 10.1038/s41467-021-22117-z (PMC7997884; doi:10.1038/s41467-021-22117-z)
Supplement: Supplementary file 5 — Supplementary Data 2 [file 41467_2021_22117_MOESM5_ESM.docx]

**Supplementary Data 2**

| **iPSC line** | **Organoid stage (days from EBs)** | **1) Developmental score**  **2) Number of neurogenic zones per organoid** | **Experiments** | **Number of organoids (starting number of EBs)** | **Comments** |
| --- | --- | --- | --- | --- | --- |
| S1 | 30 | 1) ++  2) 1-3 | qPCR, IS | 45 |  |
| C1 | 30 | 1) ++++  2) 7-8 | qPCR, IS | 45 |  |
|  | 90 | 1) ++++  2) 7-8 | scRNAseq, total RNAseq | 45 |  |
| S2 | 40 | 1) +  2) 0 | IS, size | 45 | 70% of experiments failed at early EB stage.  30% of EBs developed into organoids. |
|  | 60 | 1) +  2) 0 | Nanostring | 90 |  |
|  | 90 | 1) +  2) 0 | IS, scRNAseq, total RNAseq | 90 |  |
| S2_Corr1 | 40 | 1) +++  2) 5-7 | IS, size | 45 | 80% of EBs developed into organoids. |
|  | 60 | 1) +++  2) 5-7 | scRNAseq | 90 |  |
|  | 90 | 1) +++  2) 5-7 | IS, scRNAseq, total RNAseq | 90 |  |

**Legend:** EB = embryoid body; IS = immunostaining; scRNAseq = single-cell RNA-sequencing

**Developmental score:** + very poor/no significant development; ++ poor development; +++ modest development; ++++ normal development
