# Supplementary material for: Defective metabolic programming impairs early neuronal morphogenesis in neural cultures and an organoid model of Leigh syndrome
Source: Nat Commun. 2021 Mar 26;12:1929. doi: 10.1038/s41467-021-22117-z (PMC7997884; doi:10.1038/s41467-021-22117-z)
Supplement: Supplementary file 17 — Reporting Summary [file 41467_2021_22117_MOESM17_ESM.pdf]

nature research

Corresponding author(s): Alessandro Prigione
Last updated by author(s): 03/02/2021

Reporting Summary

Nature Research wishes to improve the reproducibility of the work that we publish. This form provides structure for consistency and transparency in reporting. For further information on Nature Research policies, see our Editorial Policies and the Editorial Policy Checklist.

Statistics

For all statistical analyses, confirm that the following items are present in the figure legend, table legend, main text, or Methods section.

n/a	Confirmed	
☐	☒	The exact sample size (*n*) for each experimental group/condition, given as a discrete number and unit of measurement
☐	☒	A statement on whether measurements were taken from distinct samples or whether the same sample was measured repeatedly
☐	☒	The statistical test(s) used AND whether they are one- or two-sided<br>*Only common tests should be described solely by name; describe more complex techniques in the Methods section.*
☒	☐	A description of all covariates tested
☒	☐	A description of any assumptions or corrections, such as tests of normality and adjustment for multiple comparisons
☐	☒	A full description of the statistical parameters including central tendency (e.g. means) or other basic estimates (e.g. regression coefficient) AND variation (e.g. standard deviation) or associated estimates of uncertainty (e.g. confidence intervals)
☐	☒	For null hypothesis testing, the test statistic (e.g. *F*, *t*, *r*) with confidence intervals, effect sizes, degrees of freedom and *P* value noted<br>*Give P values as exact values whenever suitable.*
☒	☐	For Bayesian analysis, information on the choice of priors and Markov chain Monte Carlo settings
☒	☐	For hierarchical and complex designs, identification of the appropriate level for tests and full reporting of outcomes
☒	☐	Estimates of effect sizes (e.g. Cohen's *d*, Pearson's *r*), indicating how they were calculated

Our web collection on statistics for biologists contains articles on many of the points above.

Software and code

Policy information about availability of computer code

Data collection	Whole-genome sequencing (WGS); Droplet-based single-cell RNA-sequencing; Mass-spectrometry (MS)-based proteomics and metabolomics analysis, COX enzyme activity, immunoblots, images acquisition, HCA, bioenenergetics. No software were used for other data collection.
Data analysis	We analyzed data using GraphPad Prism 8.0 (GraphPad Software, Inc.) and employed R environment for statistical computing. We assessed statistical significance using parametric tests (Student's t-test, ANOVA) for normally-distributed data and non-parametric tests (Mann-Whitney U test, Kruskal-Wallis) when normal distribution could not be verified. P values below 0.05 were considered significant.<br>For WGS analysis of off-target effects of CRISPR/eCas9 genome editing, we used the CrispRGold 1.1 algorithm (https://crisprgold.mdc-berlin.de/).<br>For scRNA-seq, we processed raw paired-end using Dropseq tools v. 2.3.0 with default parameters (https://github.com/broadinstitute/Drop-seq/releases/tag/v2.3.0). We performed alignment to the hg19 reference genome using STAR v. 2.6.0. We used Seurat v. 3.1.0 for downstream computational analyses.<br>For quantification of COX enzyme activity, we visualized stained cryosections with Leica DMI6000 microscope and took the pictures using a the software Motic Images Plus 2.0. We analyzed the images using the software MBF ImageJ bundle (formerly WCIF ImageJ).<br>Immunoblots were analyzed using Image LabTM software, version 6.0.1.<br>For bulk RNA-seq, we mapped all raw sequencing reads to the human genome (GRCh38 assembly) using STAR (version 2.6.0c) aligner. We counted reads using the htseq-count tool, version 0.9.1, with gene annotation from GENCODE release 27. We performed differential gene expression analysis using the DESeq2 (version 1.20.00) R package. We performed functional enrichment analysis using the gProfileR R package version 0.6.6, with default settings.<br>We processed raw MS data with MaxQuant software (v1.6.0.1) and searched against the human proteome database UniProtKB with 21,074 entries. We performed the correlation analysis of biological replicates and the calculation of significantly different metabolites and proteins using Perseus (v1.6.5.0).). For comprehensive proteome data analyses, we applied gene set enrichment analysis (GSEA, v2.2.3). We performed peak integration with MultiQuantTM software v.2.1.1 (Sciex, Foster City, CA).<br>We performed the integration of total RNA-sequencing, proteomics, and metabolomics using xMWAS (https://rdrr.io/github/kuppal2/

xMWAS/).

For manuscripts utilizing custom algorithms or software that are central to the research but not yet described in published literature, software must be made available to editors and reviewers. We strongly encourage code deposition in a community repository (e.g. GitHub). See the Nature Research guidelines for submitting code & software for further information.

Data

Policy information about availability of data

All manuscripts must include a data availability statement. This statement should provide the following information, where applicable:

- Accession codes, unique identifiers, or web links for publicly available datasets
- A list of figures that have associated raw data
- A description of any restrictions on data availability

The datasets generated during this study are available:
- Single-cell RNA-sequencing dataset: deposited in Gene Expression Omnibus (GEO) database; accession number: GSE152915.
- RNA-sequencing dataset: deposited in GEO database; accession number: GSE126360.
- Proteomics dataset: deposited in the ProteomeXchange Consortium server; accession: PXD019112.
- Metabolomics dataset: deposited in PeptideAtlas; identifier: PASS01598.

In accordance with the German privacy protection laws, we could not deposit the WGS genomic datasets on open repositories.

Field-specific reporting

Please select the one below that is the best fit for your research. If you are not sure, read the appropriate sections before making your selection.

☒ Life sciences ☐ Behavioural & social sciences ☐ Ecological, evolutionary & environmental sciences

For a reference copy of the document with all sections, see nature.com/documents/nr-reporting-summary-flat.pdf

Life sciences study design

All studies must disclose on these points even when the disclosure is negative.

Sample size	We chose the sample size based on previous experience and in accordance to the standards in the field. We used independent control lines and isogenic control lines to increase the robustness of the results. We performed all in vitro experiments using at least three biological replicates over different independent experiments.
Data exclusions	No data was excluded from the analyses. We performed outlier test analysis to identity potentail outliers using GraphPad: https://www.graphpad.com/quickcalcs/Grubbs1.cfm.
Replication	We repeated all experiments using at least three biological replicates over distinct independent experiments. We specified the number of biological replicates and independent experiments in the respective figure legends.
Randomization	We plated the cells in a random distribution onto cell culture and multi-well plate positions, and randomly assigned them to experimental groups. We perfomed cell counting on random miscroscope view fields. We then grouped the samples based on their genotypes or the specific treatments, and we compared them to the respective control groups.
Blinding	The investigators who performed the RNA sequencing, proteomics, metabolomis, omics integration analysis, and COX activity staining were blinded to the genotypes of groups and samples.

Reporting for specific materials, systems and methods

We require information from authors about some types of materials, experimental systems and methods used in many studies. Here, indicate whether each material, system or method listed is relevant to your study. If you are not sure if a list item applies to your research, read the appropriate section before selecting a response.

Materials & experimental systems

n/a	Involved in the study	
☐	☒	Antibodies
☐	☒	Eukaryotic cell lines
☒	☐	Palaeontology and archaeology
☒	☐	Animals and other organisms
☒	☐	Human research participants
☒	☐	Clinical data
☒	☐	Dual use research of concern

Methods

n/a	Involved in the study	
☒	☐	ChIP-seq
☒	☐	Flow cytometry
☒	☐	MRI-based neuroimaging

Antibodies

Antibodies used	For immunostaining: PAX6 (Covance, 1:200), SOX2 (Santa Cruz, 1:100), TUJ-1 (Sigma-Aldrich, 1:3000), LIN28 (ProteinTech Europe, 1:300), TRA-1-60 (Millipore, 1:200), MAP2 (Synaptic System, 1:100), GFAP (Synaptic Systems, 1:500), NANOG (R&D Systems, 1:200), smooth muscle actin (SMA) (DakoCytomation, 1:200), SOX17 (R&D Systems, 1:50), TH (Millipore, 1:300), FOXA2 (Sevenhills, 1:100), S100ß (Abcam, 1:500), SYP (Sigma-Aldrich, 1:500), SYN (Invitrogen #7H10G6, 1:100), VAMP2 (Synaptic systems, 1:500), NESTIN (Millipore, MAB5326, 1:200), NURR1 (Sigma-Aldrich, 1:500), pVIM (MBL Life Science, 1:1000). Corresponding secondary antibodies Alexa Fluor (1:2000, Life Technologies).<br>For MACS: PSA-NCAM-PE (Miltenyi Biotec, #130–093-274).<br>For immunoblotting: MT-CO2 (ab110258 from Abcam, 1:1,500), beta-actin (ACTB from Sigma, 1:4,000), PGC1A (Novus Biological, NBP1-04676, 1:0000), GAPDH (Immunological Sciences, MAB-10578, 1:0000), OXPHOS rodent antibody cocktail (Abcam, #ab110413, composed by a cocktail of 5 antibodies: SDHB, UQCRC2, MT-CO1, ATP5A, and NDUFB8), COX4I1 (Invitrogen, A21347), and SDHA (Abcam, ab14715).
Validation	All antibodies used in this study are commercial and well-established in the field. Validation data are provided for each antibody by the manufacturer. In addition, we validated the antibodies for staining, MACS, and immunoblotting using hESC-derived neural cells and using autopsy-derived brain slices.

Eukaryotic cell lines

Policy information about cell lines

Cell line source(s)	We purchased the human embryonic stem cell (hESC) line H1 from WiCell and used it in accordance with the German license issued to Prof. Alessandro Prigione by the Robert Koch Institute (AZ: 3.04.02/0077-E01).<br>Control iPSC lines were all previously generated. Dr. Heiko Lickert (Helmholtz Center Munich) kindly provided CTL iPSC line C1, which was previously named XM001 and was obtained from a 47 years-old healthy woman using episomal plasmids. The MDC Stem Cell Core facility kindly provided CTL iPSC line C2, which was generated by SCVI Stanford Cardiovascular Institute using Sendai viruses and was originally named SCVI113. We previously generated CTL iPSC line C3, which we derived from healthy neonatal foreskin fibroblasts using episomal plasmids and we previously named TFBJ.<br>Patient iPSC lines were generated for this study after signed and informed patient consent. Patient S1 was male and carried an homozygous SURF1 mutation c.530T>G p.(V177G). Patient S2 was male and carried the homozygous SURF1 mutation c.769G>A p.(G180R). The SURF1 study was approved by the IRB of the Charité (EA2/131/13 and EA2/107/14). Patient NDU_1 was male and carried the NDUFS4 mutation c.462delA p.(K154fs). Patient NDU_2 was female and carried the NDUFS4 mutation c.316C>T p.(R106*). The NDUFS4 study was approved by Technical University Muenchen (#5360/12 S).<br>ll details regarding the PSC lines used (age, source, reprogramming method) are reported in Supp Table 13.
Authentication	We authenticated all iPSC lines (from control subjects, patients, and genetically modified) using Sanger sequencing and SNP karyotyping (Infinium OmniExpressExome-8 Kit from Illumina). hESCs were authenticated by the provider (WiCell).
Mycoplasma contamination	We routinely monitored against mycoplasma contamination using PCR. All samples used in this study tested negative for mycoplasma contamination.
Commonly misidentified lines<br>(See ICLAC register)	No commonly misidentified lines were used.
